# Biological Activity of N-Hydroxyethyl-4-aza-2,3-didehydropodophyllotoxin Derivatives upon Colorectal Adenocarcinoma Cells

**DOI:** 10.4236/ojmc.2014.41001

**Published:** 2014-03

**Authors:** Christian Vélez, Beatriz Zayas, Ajay Kumar

**Affiliations:** Universidad Metropolitana, School of Environmental Affairs San Juan, Puerto Rico, USA

**Keywords:** Etoposide, Podophyllotoxin, Aza-Podophyllotoxin, Colon Cancer, COLO 205

## Abstract

Etoposide is a chemotherapy drug derived from the natural lignin podophyllotoxin. Our novel generated Aza-podophyllotoxin compounds (AZP 8a & AZP 9a) are analogues of podophyllotoxin and were previously screened for anti-cancer activity through the NCI 60 cell line screening panel showing activity on various cell types including colon cancer. This study expands the toxicological screening by studying apoptosis and various hallmark events as part of the mechanism of action of these compounds on colon cancer cells. The COLO 205 cell line was selected and exposed to AZP to determine the IC50 doses at 24 hours treatment. Apoptosis hallmark events such as migration of phosphatidylserine (PS) to the cell membrane, DNA fragmentation, cell cycle effects, mitochondrial membrane permeabilization and caspase activation were included. Experiments were performed in triplicates for all tested compounds including AZP 8a, AZP 9a, camptothecin as positive control and vehicle as negative control. Our results present contrasting apoptotic activity between the experimental compounds. Compound 8a presented migration of PS (annexin V assay), DNA fragmentation and cell cycle arrest at S phase. Compound 9a presented PS migration with fragmented DNA, cell cycle arrest at S phase, mitochondrial membrane permeabilization and activation of caspase 3, 8 and 9. Compound 8a without the oxygen atoms in ring A appears to cause effects similarly to autophagy as induced by etoposide, a cancer drug analogue of our heterocyclic compounds. Compound 9a with the oxygen atoms in expanded ring A presented induction of cell death following activation of a classical apoptosis pathway. Our results suggest that minor structural differences among these AZP can account for the difference in biological response and cancer cell toxicity.

## 1. Introduction

**Figure 1:**
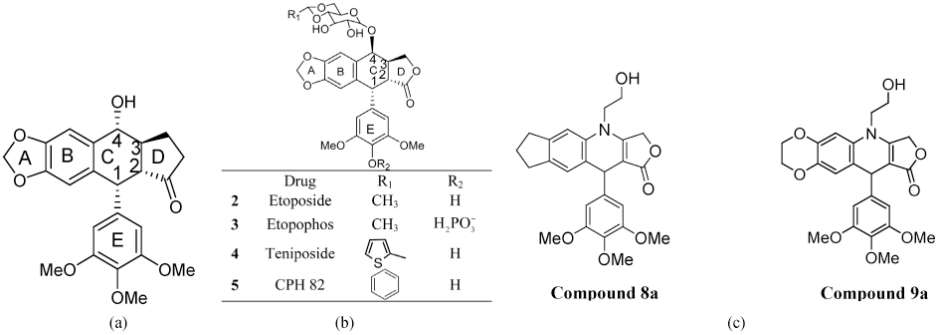
(a) Structure of Podophyllotoxin 1; (b) Drugs Derived from Podophy llotoxin; (c) N-Hydroxyethyl-4-aza-didehyropodophyllotoxin Derivatives.

The American mayapple (*Podophyllum*) has been a source of bio-active lignans for many years. The most studied one of these is podophyllotoxin (**1**) Figure 1(a).

This natural compound **1** presents a chemical structure consisting of four fused planar rings with consecutive chiral centers [1]. Original uses for podophyllotoxin since 1942 (still in use) include topical applications [2]. Among the various medical applications of podophyllotoxin or its derivatives are skin disorders, periodontal disease, parasitic infections and a number of anti-viral applications among others [3]. Podophyllotoxin was antimitotic and tubulin inhibitor by itself [4] but later it was developed as a topoisomerase-II inhibitor. Several semisynthetic derivatives of podophyllotoxin have been used as chemotherapeutic drugs. These current derivatives are etoposide (**2**), etopophos (**3**) and teniposide (**4**) [5] (Figure 1(b)).

Arguably the most important derivative of these has been etoposide (also known as VP-16). This drug has been widely studied and has been classified since the 1980's as a topoisomerase II inhibitor [6].

The success of etoposide has led to a great deal of work with podophyllotoxin derivatives, much of it studying a variety of biological effects and anti-cancer activities [7] [8].

Synthesis of podophyllotoxin in laboratory is still quite challenging chemistry [9]. To fulfill the industrial demand of **1** we are still dependent on the endangered natural sources of this compound. **1** is also the precursor to a new derivative CPH 82 (**5**) (Figure 1(c)) which has been tested for rheumatoid arthritis in Europe [10], and it is the precursor to other derivatives used for the treatment of psoriasis [11] and malaria.

In our previous work we explored the synthesis and application of novel aza-podophyllotoxin derivative compounds which led to several new analogs with anti-cancer potential [12]. Using a one pot method, synthesis of an aza-podophylltoxin derivative is less complicated and results in a high yield. This method also, allows us to create extensively modified libraries of several novel derivatives, which were previously impossible to synthesize from podophyllotoxin [13]. These new aza-podophyllotoxin derivative compounds have only two chiral centers at the 1 and 4 positions of ring “C” as compared to 4 chiral centers in ring “C” of podophyllotoxin. Elimination of chirality at C2 and C3 by double bond in aza-podophyllotoxin is helping all the 4 A, B, C, and D fused rings in one plane, better than podophyllotoxin. Probably this is one of the reasons for enhanced activity of aza-podophyllotoxin derivatives against tumor cells [14]. Most of the drugs from our library showed excellent activity against 60 types of human cancer cell line panels performed at the United States National Cancer Institute (NCI) [15]. Two AZP derivatives 8a (4-(2-Hydroxyethyl)-10-(3,4,5-trimethoxyphenyl)-3,4,6,7,8,10-hexahydro-1H-yclopenta[g]furo[3,4-b]quinolin-1-one) (Figure 1(c)) and 9a (6-(2-Hydroxyethyl)-10-(3,4,5-trime-thoxyphenyl)-2,3,7,10-tetrahydro-[1,4]dioxino[2,3-g]furo[3,4-b]quinolin-9(6H)-one) (Figure 1(c)) were found very active against COLO 205 colon cancer cell line. Compound 8a does not contain an oxygen atom in ring A, while compound 9a contains 2 oxygen atoms and expanded ring with 1 carbon in ring A. Here we are presenting the major apoptotic associated activities of 8a and 9a in COLO 205 human cancer cell line directed to determine the mechanism of action of these novel podophyllotoxin derivatives. This information will establish the background biological activities necessary for conducting further *in vivo* and gene expression studies.

## 2. Materials and Methods

### 2.1. Cell Culture

The cell line used in this study was the COLO-205 human colorectal adenocarcinoma (ATCC CCL-222). Cells were maintained in RPMI 1640 (ATCC, Manassas VA) containing 10% fetal bovine serum (ATCC). Cultures were maintained at 37°C with humidified atmosphere of 95% air/5% CO_2_. Measurement of apoptosis endpoints was determined at a 24 hours exposure. This time of exposure allows sufficient time to screen various events that occur within 24 hours [15].

### 2.2. Annexin V

The annexin V assay has been used as an apoptosis event indicator thru the detection of phosphatidylserine (PS) on the exterior surface of cells, a key event in apoptotic cells [16]-[18]. Approximately 4 × 10^6^ cells were treated for 24 hours with the GI_50_ dose of each compound (80 nM of 8a and 180 nM of 9a) as previously determined [12]. Controls used in all assays were etoposide (3.38 μM), podophyllotoxin (3.44 μM) and vehicle (DMSO). After 24 hours of exposure, cells were stained with annexin V conjugate, and propidium iodide (Biotium, Hayward, CA). Samples where then analyzed using the Nucleo Counter NC3000 system (Chemometec, Allerød, Denmark). All experiments described in this section were analyzed using the Nucleo Counter NC3000 system, reagents and kits are specified by the manufacturer.

### 2.3. DNA Fragmentation

The measurement of deoxyribonucleic acid (DNA) fragmentation after exposure to chemical substances has been used as a tool for apoptosis detection for a number of years [19] [20]. This event which is mediated by nucleases can be measured using DNA content and measuring cells containing less than 1DNA equivalent known as Sub-G_1_. This assay is based on removal of small DNA fragments and the retention of 4′,6-diamidino-2-pheny-lindole (DAPI) stained fragments which have a higher weight. After 24 hours treatment with the test drugs and controls at their respective GI_50_ dose, cells fixed with ethanol 70%, stained with 1 μg/ml DAPI, and then analyzed by differential image analysis which measured DAPI intensity.

### 2.4. Cell Cycle Effects

Changes to cell cycle can provide insight to therapeutic drug's mechanism of action and targeting this key cellular event has been used as mechanism against cancer cells [21]-[23]. In order to study any possible alterations in the cell cycle, the DAPI based NC3000 fixed cell cycle assay was performed. Cells exposed to the experimental drugs were stained with 1 μg/ml DAPI and analyzed to measure DNA content.

### 2.5. Mitochondrial Membrane Permeabilization

Mitochondrial membrane permeabilization (MMP) is a well-known event during apoptosis [24]. A total of 1 × 10^6^ cells were exposed to the test compounds and controls etoposide and podophyllotoxin at previously described doses, then labeled with 200 μg/ml of 5,5′,6,6′-tetrachloro-1,1′,3,3′-tetraethylbenzimidazolocarbocyanine iodide (JC-1) stain to assess mitochondrial potential changes. A counter stain with 1 μg/ml DAPI in PBS was also applied, then analyzed to detect the fluorescent markers.

### 2.6. Caspase Activity

Caspase activation, a hallmark of the apoptosis process [25], was assessed to determine the response to the tested compounds. Cells were exposed to the compounds as described previously, harvested then stained with Fluorescent Labeled Inhibitors of Caspases (FLICA) which bind to active caspases. The green FAM FLICA kit (Immunochemistry Technologies, Bloomington Min) for caspase 3, 8 and 9 were used as per manufacturer's specifications then analyzed the fluorescent probe.

### 2.7. Statistical Analysis

A one way ANOVA and Post Hoc Test Tukey was also performed on all experiments to determine statistical significant differences.

## 3. Results

### 3.1. Annexin V

**Figure 2:**
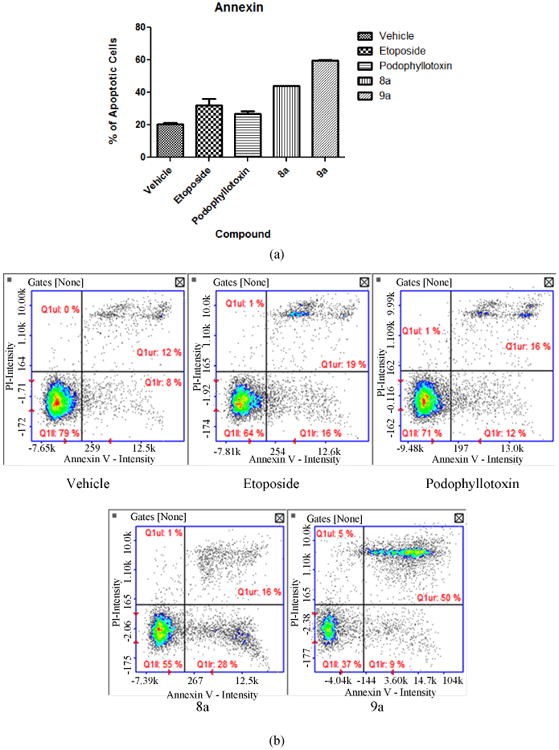
(a) Annexin V analysis. Results clearly indicate apoptotic activity for both compounds. 8a presents 44% of apoptotic cells whereas 9a presents 59.5%. Both compounds present higher activity than the positive controls etoposide (32%) and podophyllotoxin (27%); (b) Annexin V Staining Histogram. Data presents the stage of apoptosis after exposure to the tested compounds. Compound 9a presents the majority of cells (50%) at a late apoptotic stage (top right quadrant). Positive controls and test compound 8a present mostly cells at an early apoptotic stage clustered in the bottom right quadrant.

Expression of phosphatidylserine was measured as an apoptosis marker. Both tested compounds presented statistical significance (P < 0.05) for apoptosis induction. Compound 8a presented 44% of apoptotic cells while 9a presented 59.5%. Figure 2(a) presents the values obtained. Compound 9a presented a majority of cells at a late apoptotic stage (50%) shown on Figure 2(b). Both compounds are comparable to the positive controls etoposide (32%) and podophyllotoxin (27%) clearly showing apoptotic activity.

### 3.2. DNA Fragmentation

**Figure 3:**
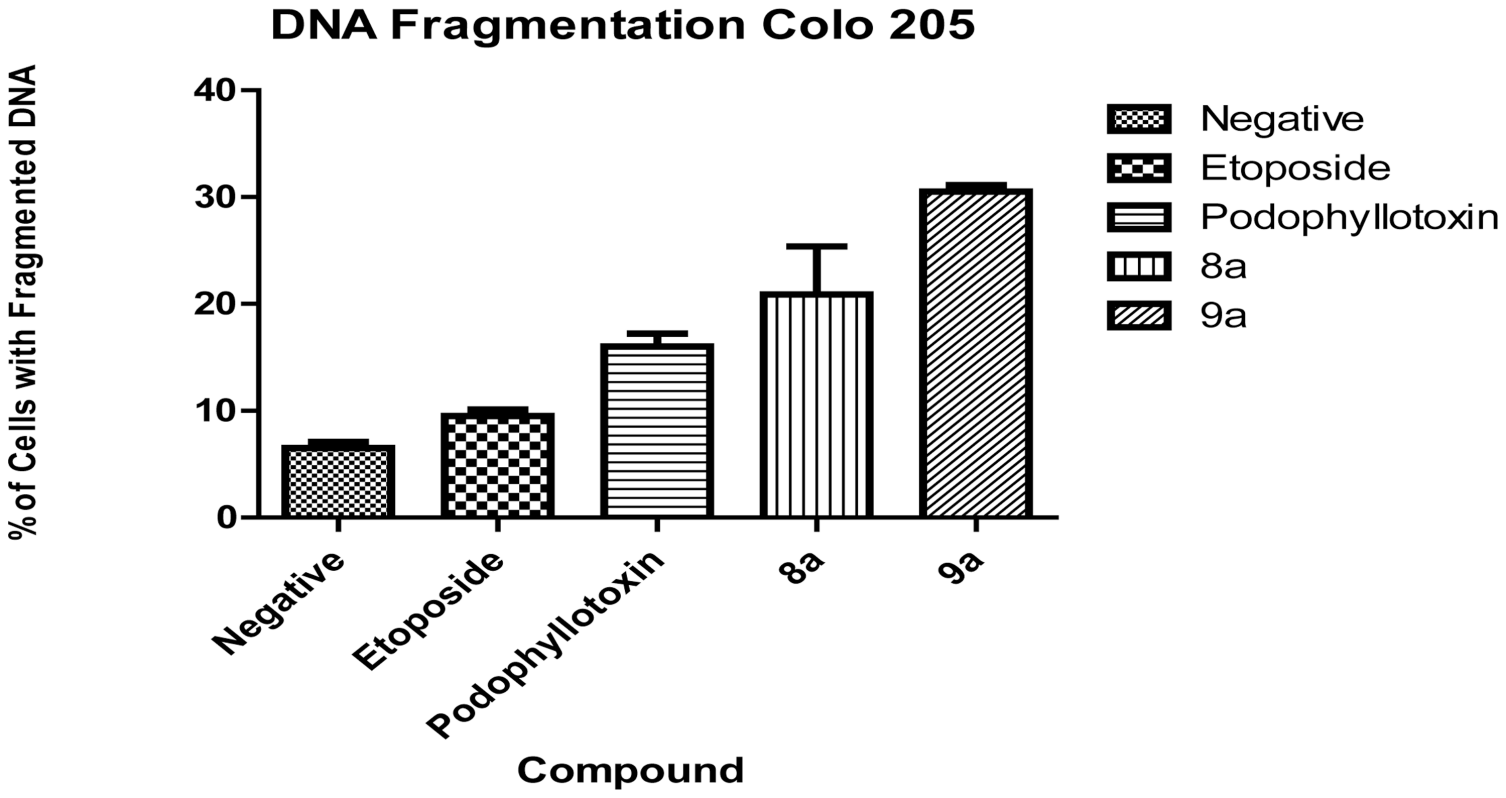
DNA Fragmentation. Results indicate significant DNA fragmentation for both tested compounds 8a (20.9%) and 9a (30.5%) in comparison with the analog controls etoposide (9.5%) and podophyllotoxin (16%).

Cells with fragmented DNA were assessed using DAPI staining. Results indicate significant fragmentation for both tested compounds. Figure 3 shows results for DNA fragmentation, 8a presented a mean of 20.9% DNA fragmentation whereas 9a presented 30.5%. These results contrast with the structural analog etoposide (9.5%) and podophyllotoxin (16%) which did not cause statistically significant DNA fragmentation.

### 3.3. Cell Cycle Effects

**Figure 4:**
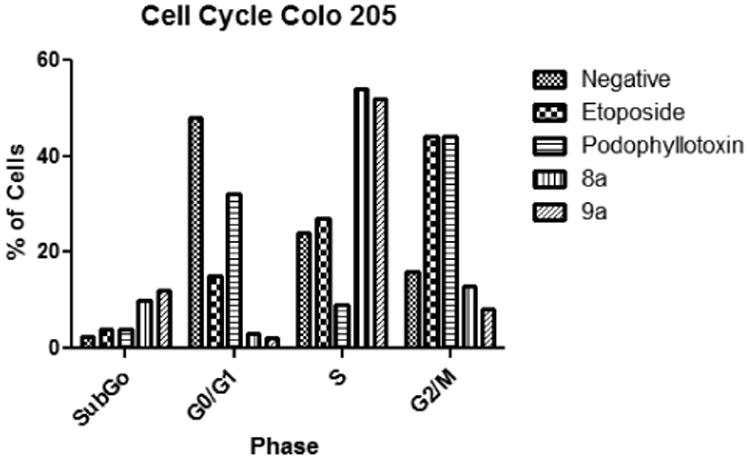
Cell Cycle. Results indicate that the majority of cells exposed to the tested compounds were arrested at the S phase. The majority of the cells exposed to compound 8a were arrested at the S phase followed by 13% at the G2/M, 10% at the Sub G0 and 3% at the G0/G1 stage. For compound 9a the majority of cells (52%) were arrested at the S phase followed by 12% at the sub G0 stage, 8% at the G2/M and 2% at the G0/G1 stage.

We analyzed the cell cycle to assess possible details which may give insight to the mechanism of action of the tested compounds. Our results indicate that the majority of cells exposed to the tested compounds were arrested at the S phase of the cell cycle (Figure 4). A total of 54% of cells exposed to compound 8a were arrested at the S phase followed by 13% at the G2/M, 10% at the Sub G0 and 3% at the G0/G1 stage. For compound 9a the majority of cells (52%) were arrested at the S phase followed by 12% at the sub G0 stage, 8% at the G2/M and 2% at the G0/G1 stage.

### 3.4. Mitochondrial Membrane Permeabilization

**Figure 5:**
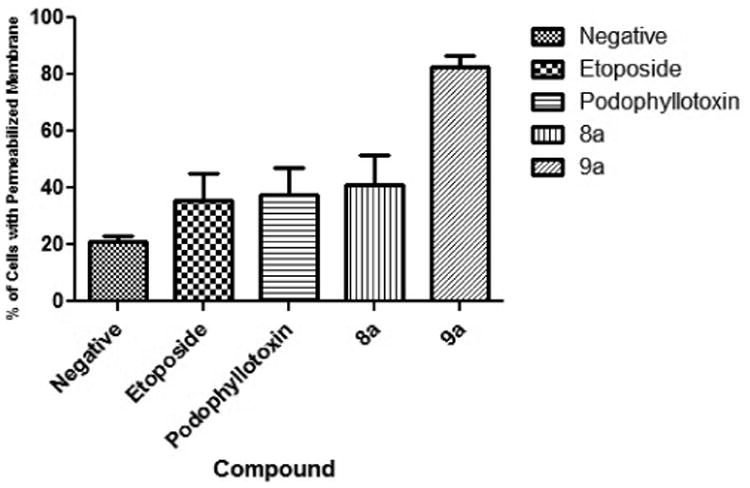
Mitochondrial Membrane Permeabilization. Results indicate that compound 9a caused significant mitochondrial permeabilization, but not 8a. Compound 9a caused a mean of 82.5%, compound 8a 41%, the controls etoposide 35.5%, podophyllotoxin 37.5% and the vehicle 21% mitochondrial membrane permeabilization.

Mitochondrial mediated apoptosis can be an indicator of intrinsic apoptotic pathway. We examined this phenomenon to assess the possibility of an intrinsic mechanism after exposure to our tested compounds. Our results indicate that only compound 9a induced a statistically significant (P < 0.05) permeabilization of the mitochondrial membrane (MM). Compound 9a caused a mean of 82.5% (Figure 5) of cells with permeabilized MM while compound 8a caused 41% which was not statistically significant and comparable to the compounds which also did not cause MM permeabilization.

### 3.5. Caspase 3 y 7 Activation

**Figure 6:**
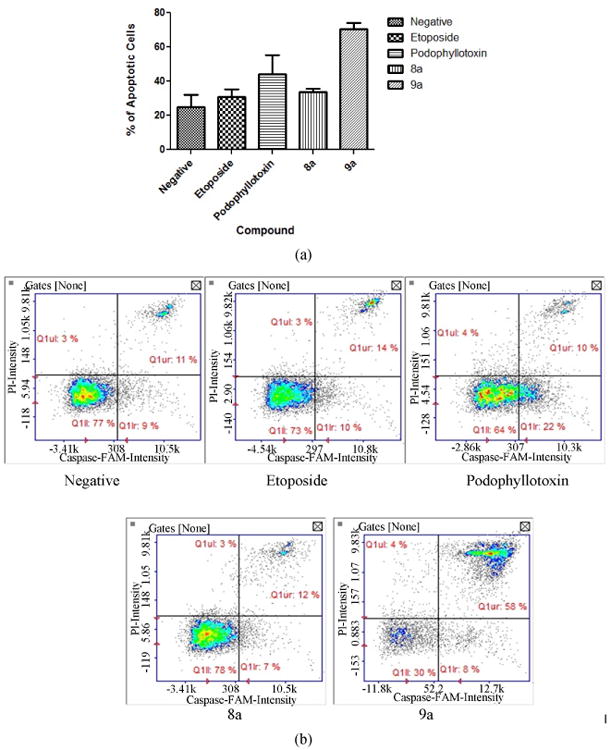
(a) Caspase 3 and 7 activation. Results reveal that among the experimental drugs compound 9a caused significant activation of effector caspases in contrast to the compound 8a; (b) Caspase 3 and 7 activation. Compound 9a caused significant activation of effector caspases in contrast to compound 8a. As observed the majority of cells are in the late apoptotic stage (upper right quadrant). Caspase activated green fluorescent cells (lower right box) were used in this assay.

Caspase activation can provide insight in the mechanism of action and cell death processes. We analyzed caspase 3 and 7 effector caspases after exposure to our tested compounds. Results show (Figure 6(a)) that only compounds 9a (70.5%) and positive control podophyllotoxin (44%) caused significant activation of effector caspases whereas compound 8a caused no significant activation (33.5%) comparable to the negative control. Interestingly, only cells exposed to compound 9a were detected at a late apoptotic stage (Figure 6(b)).

### 3.6. Caspase 8 Activation

**Figure 7:**
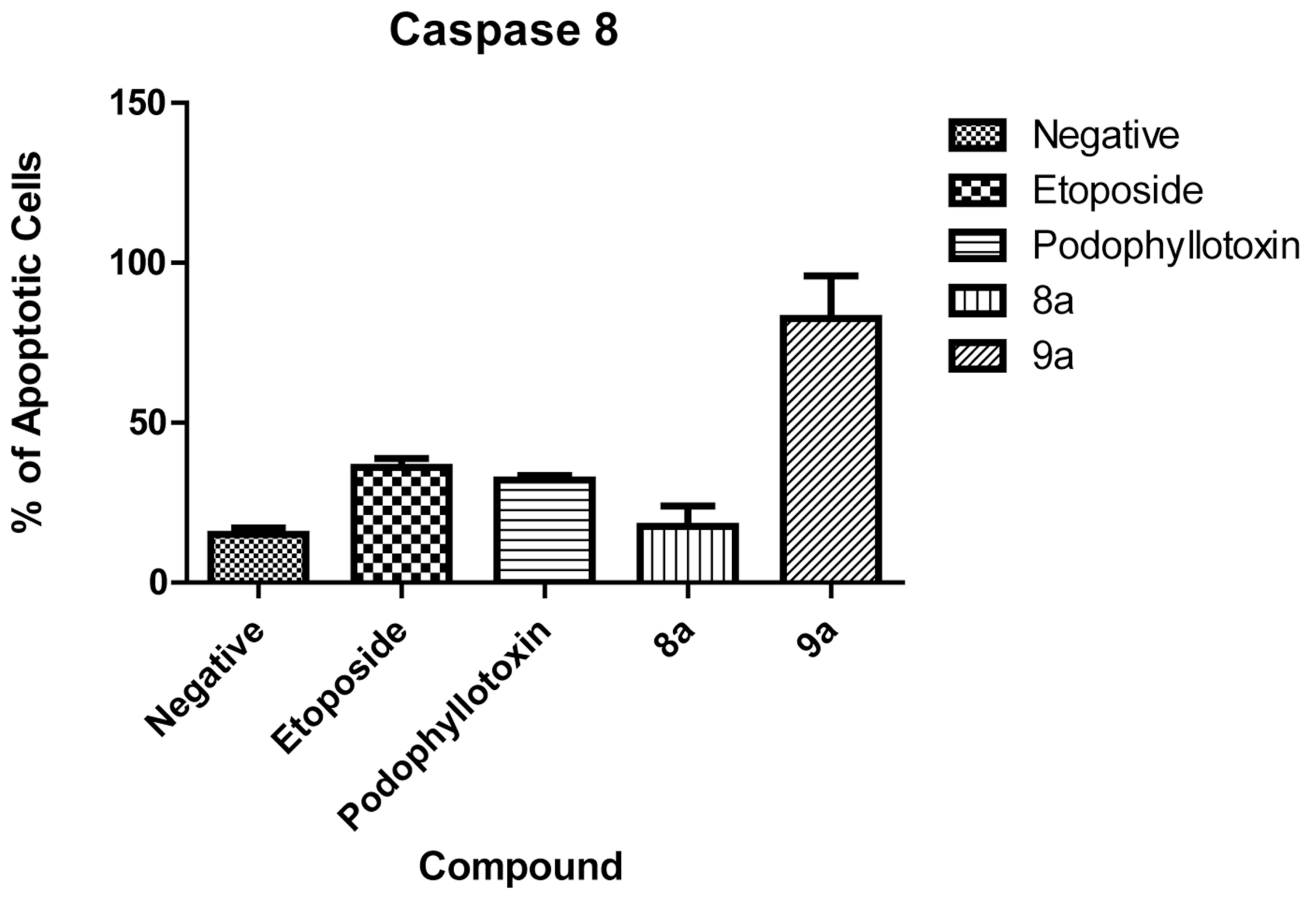
Caspase 8 activation. Results show that compound 9a activated caspase 8 on treated cells in contrast to the compound 8a where no significant activity was detected.

Caspase 8 was measured to determine if apoptosis occurs via an intrinsic or extrinsic pathway. Our results show that only compound 9a caused significant activation of caspase 8 with an 82.5% of cells with activated caspase 8 (Figure 7). These caspase activated cells were again detected in the late apoptotic stage as in the caspase 3 results. All other compounds did not present significant activation of caspase 8.

### 3.7. Caspase 9 Activation

**Figure 8:**
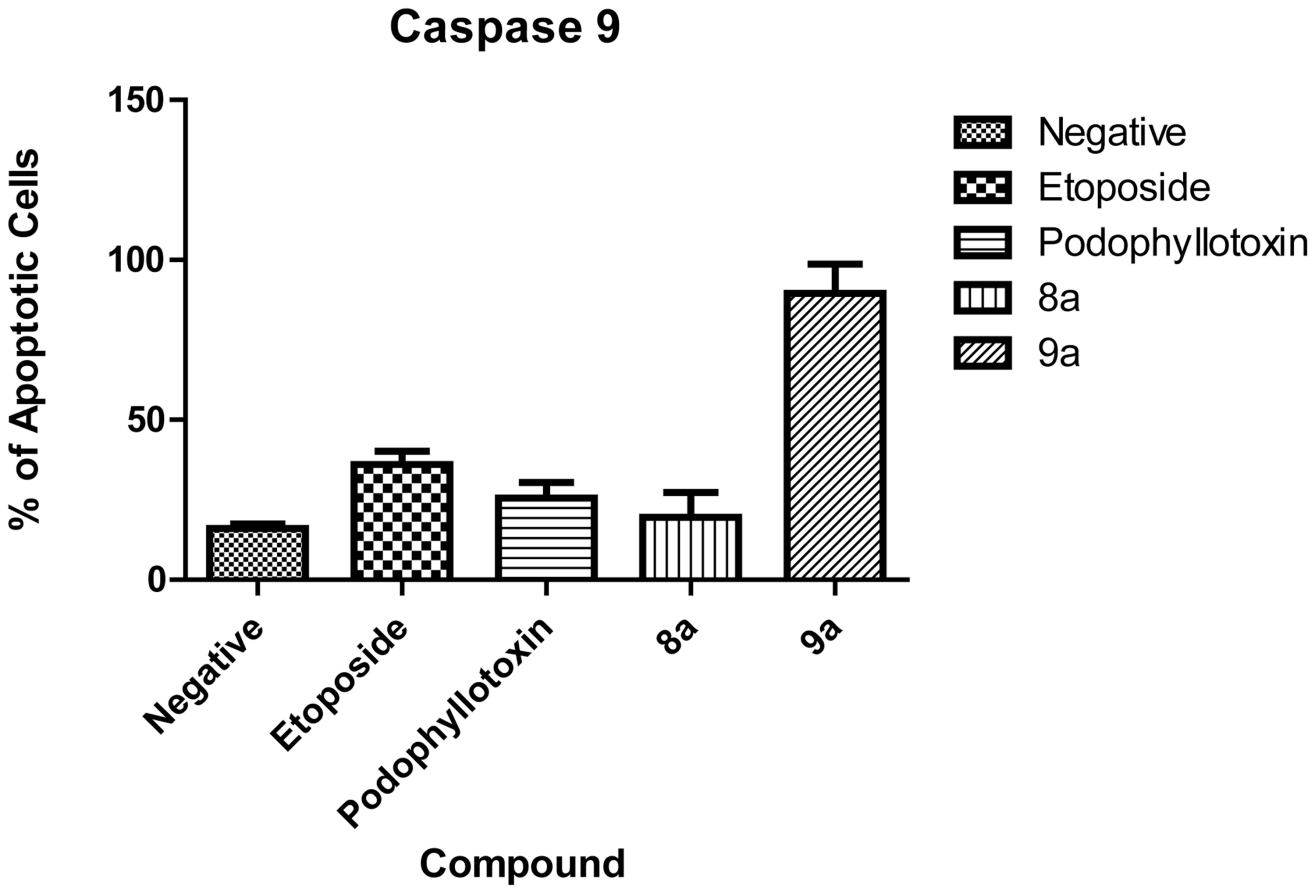
Caspase 9 activation. when comparing our test drugs, only compound 9a induced significant activation of caspase 9 in contrast to compound 8a.

Caspase 9 activation is a hallmark event of the intrinsic apoptotic pathway. Our results show activation of caspase 9 on cells treated with the 9a compound (89.5%). This was unexpected since caspase 8 was also activated thus an extrinsic mechanism was presumed to be involved (Figure 8).

## 4. Discussion

In this study we screened the biological activity and apoptosis induction of two novel aza-podophyllotoxin derivatives. These compounds were previously assayed for toxicity against a number of cell lines and were determined to be active for growth inhibition upon COLO 205 colorectal adenocarcinoma cells among other cancer cell types. Both of our tested compounds presented different biological activities on the COLO 205 cell line. Compound 8a presented an unexpected apoptotic activity profile. This compound presented PS migration to the exterior of cells as evidenced by the annexin V staining. Migration of PS is usually indicative of an apoptotic cell death mechanism. Additionally this compound presented a significant cell cycle arrest at the S phase and as well as DNA fragmentation; however none of the traditional apoptosis hallmarks such as mitochondrial membrane permeabilization and caspase 8, 9, 3 and 7 activation were detected in a significant manner.

This type of apoptosis sometimes referred to as atypical apoptosis, has been observed after activation of intra-S DNA damage checkpoints [26], or p27 mediated autophagy [27]. Intra-S checkpoint effects involve the phosphorylation of proteins such as the retinoblastoma tumor suppression protein (pRB) causing accumulation of cells at the S phase to permit reparation of any DNA damage. P27 mediated autophagy results from inhibition of cyclin dependent kinases (CDK) which regulate cell cycle progression [28] [29]. Other types of similar effects include inhibition of the topoisomerases in which cell cycle arrest with DNA fragmentation have been documented [30] in this case however, evidence of caspase 3 activation was observed. Given our compounds close structural relationship with the known topoisomerase II inhibitor, etoposide; we deduced that biological activities could be similar to this chemotherapeutic drug.

Our results for compound 8a are similar to previous work [31] in which etoposide induces autophagic cell death through a BCL-2 dependent mechanism. The activity of compound 9a included the same positive parameters as in compound 8a such as PS migration, DNA fragmentation and cell cycle arrest at S phase. This compound however did cause activation of caspases 3, 8 and 9 in a significant manner. Generally the type of activated caspase can give insight to the cell death mechanism that is occurring. The death receptor pathway (also known as extrinsic) is usually associated with activation of caspase 8 [32]. Other evidence exists in which this caspase can be activated by other mechanisms such as p53 [33]. Intrinsic mediated pathways include the liberation of cytochrome C after mitochondrial membrane permeabilization. This pathway can cause activation of the apoptosome complex involving caspase 9 [34]. It is this caspase in its activated form that cleaves effector caspase 3 which acts upon numerous cell substrates that can cause the characteristic DNAse mediated fragmentation [35] [36]. If we consider this information in the context of compound 9a we can see that the classical apoptosis effects are present. Work by Liu *et al.* [37] demonstrates that etoposide can cause p53/p73 apoptosis through a caspase 8 signal amplification of the intrinsic mitochondrial pathway. This suggests a similar mechanism for 9a in which we evidenced mitochondrial permeability as well as caspase activation including the classic hallmarks of apoptotic cell death.

## 5. Conclusion

Although a complete mechanistic description was beyond the scope of this work, we found interesting evidence which suggests a structure-activity difference for our aza-podophyllotoxin derivatives. Compound 8a presents similar core structure to the podophyllotoxin, precursor of etoposide. The only difference is substitution of nitrogen in ring “C” at position 4, elimination of two chiral centers and elimination of 2 oxygen atoms from ring A. Substitution of nitrogen in ring C might not make significant difference in biological activity [38] and elimination of chirality from ring C probably enhancing its biological activity by increasing planarity in one plane of all four fused rings [39] [40]. Elimination of 2 oxygen atoms from ring A is probably playing a key role in the biological activity of these compounds. 8a does not have the two oxygen atoms in ring A appearing to have activity similar to etoposide inducing autophagy [31], whereas compound 9a has minor structural difference from compound 8a except that it has two oxygens and ethylene group on ring A presented typical apoptosis cell death with the hallmarks of etoposide induced apoptosis. Overall, this preliminary biological screening for these novel substances provides the ground work for further insight on these promising substances.

## References

[1] Nagar N, Jat R, Saharan R, Verma S, Sharma D, Bansal K (2011). Podophyllotoxin and Their Glicosidic Derivatives. Pharmacophore.

[2] Kaplan IW (1942). Codylomata acuminata. The New Orleans Medical and Surgical Journal.

[3] Liu YQ, Yang LM, Tian X (2007). Podophyllotoxin: Current Perspectives. Current Bioactive Compounds.

[4] Wang RW, Rebhum LI, Kupchan SM (1977). Anti Mitotic and Antitubulin Activity of the Tumor Inhibitor Steganacin. Cancer Research.

[5] Stähelin HF, Von Wartburg A, Stã HF (1991). The Chemical and Biological Route from Podophyllotoxin Glucoside to Etoposide: Ninth Cain Memorial Award Lecture The Chemical and Biological Route from Podophyllotoxin Glucoside to Etoposide. Ninth Cain Memorial Award Lecture.

[6] Baldwin EL, Osheroff N (2005). Etoposide, Topoisomerase II and Cancer. Current Medicinal Chemistry —Anti-Cancer Agents.

[7] Liu J, Cao B, Gao Y, Bai M, Mei X, Chen H, Jiang YG, Huang DJ (2013). Design, Synthesis, and Anti-tumor Activity of Novel Podophyllotoxin Derivatives as Potent Anticancer Agents. Journal of Asian Natural Products Research.

[8] Ma Y, Fang S, Li H, Han C, Lu Y, Zhao Y, Liu Y, Zhao C (2013). Biological Evaluation and Molecular Modelling Study Of podophyllotoxin Derivatives as Potent Inhibitors of Tubulin Polymerization. Chemical Biology & Drug Design.

[9] Reynolds AJ, Scott AJ, Turner CI, Sherburn MS (2003). The Intramolecular Carboxyarylation Approach to Podophyllotoxin. Journal of the American Chemical Society.

[10] Larsen A, Petersson I, Svensson B (1989). Podophyllum Derivatives (CPH 82) Compared with Placebo in the Treatment of Rheumatoid Arthritis. British Journal of Rheumatology.

[11] Wantke F, Fleischl G, Götz M, Jarisch R (1993). Topical Podophyllotoxin in Psoriasis Vulgaris. Dermatology.

[12] Kumar A, Kumar V, Alegria A, Malhotra S (2011). N-Hydroxyethyl-4-aza-didehydropodophyllo Toxin Derivatives as Potential Antitumor Agents. European Journal of Pharmaceutical Sciences.

[13] Kumar A, Alegria AE (2010). Synthesis of Novel Functionalized 4-aza-2,3-didehydropodophyllotoxin Derivatives with Potential Anti-Tumor Activity. Journal of Heterocyclic Chemistry.

[14] Hitotsuyanagi Y, Fukuyo M, Tsuda K, Kobayashi M, Ozeki A, Itokawa H, Takeya K (2000). 4-Aza-2,3-dehydro-4-deoxypodophyllotoxins: Simple Aza-Podophyllotoxin Analogues Possessing Potent Cytotoxicity. Bioorganic & Medicinal Chemistry Letters.

[15] Kravtsov VD, Daniel TO, Koury MJ (1999). Comparative Analysis of Different Methodological Approaches to the *in Vitro* Study of Drug-Induced Apoptosis. The American Journal of Pathology.

[16] Huang WW, Ko SW, Tsai HY, Chung JG, Chiang JH, Chen KT, Chen YC, Chen HY, Chen YF, Yang JS (2011). Cantharidin Induces G2/M Phase Arrest and Apoptosis in Human Colorectal Cancer COLO 205 Cells through Inhibition of CDK1 Activity and Caspase-Dependent Signaling Pathways. International Journal of Oncology.

[17] Mukherjee A, Dutta S, Shanmugavel M, Mondhe DM, Sharma PR, Singh SK, Saxena AK, Sanyal U (2010). 6-Nitro-2-(3-hydroxypropyl)-1H-benz[de]isoquinol ine-1,3-dione, a Potent Antitumor Agent, Induces Cell Cycle Arrest and Apoptosis. Journal of Experimental & Clinical Cancer Research.

[18] Schutters K, Reutelingsperger C (2010). Phosphatidylserine Targeting for Diagnosis and Treatment of Human Diseases. Apoptosis: An International Journal on Programmed Cell Death.

[19] Kumar NDR, George VC, Suresh PK, Kumar RA (2012). Cytotoxicity, Apoptosis Induction and Anti-Metastatic Potential of Oroxylum indicum in Human Breast Cancer Cells. Asian Pacific Journal of Cancer Prevention: APJCP.

[20] Sharma M, Agrawal SK, Sharma PR, Chadha BS, Khosla MK, Saxena K (2010). Cytotoxic and Apoptotic Activity of Essential Oil from Ocimumviride towards COLO 205 Cells. Food and Chemical Toxicology: An International Journal Published for the British Industrial Biological Research Association.

[21] Wei PL, Tu SH, Lien HM, Chen LC, Chen CS, Wu CH, Huang CS (2012). The *in Vivo* Antitumor Effects On human COLO 205 Cancer Cells of the 4,7-Dimethoxy-5-(2-propen-1-yl)-1,3-benzodioxole (apiole) Derivative of 5-Substituted 4,7-dimethoxy-5-methyl-l,3-benzodioxole (SY-1) Isolated from the Fruiting Body of Antrodia Camphorate. Journal of Cancer Research and Therapeutics.

[22] Lin JC, Ho YS, Lee JJ, Liu CL, Yang TL, Wu CH (2007). Induction of Apoptosis and Cell-Cycle Arrest in Human Colon Cancer Cells by Meclizine. Food and Chemical Toxicology: An International Journal Published for the British Industrial Biological Research Association.

[23] Ho YS, Wu CH, Chou HM, Wang YJ, Tseng H, Chen CH, Chen LC (2005). Molecular Mechanisms of Econazole-Induced Toxicity on Human Colon Cancer Cells: G0/G1 Cell Cycle Arrest and Caspase 8-Independent Apoptotic Signaling Pathways. Food and Chemical Toxicology: An International Journal Published for the British Industrial Biological Research Association.

[24] Watanapokasin R, Jarinthanan F, Nakamura Y, Sawasjirakij N, Jaratrungtawee A, Suksamrarn S (2011). Effects of α-Mangostin on Apoptosis Induction of Human Colon Cancer. World Journal of Gastroenterology: WJG.

[25] Yi CH, Yuan J (2009). The Jekyll and Hyde Functions of Caspases. Developmental Cell.

[26] Ory B, Blanchard F, Battaglia S, Gouin F, Re F, Heymann D (2007). Zoledronic Acid Activates the DNA S-Phase Checkpoint and Induces Osteosarcoma Cell Death Characterized by Apoptosis-Inducing Factor and Endonuclease-G Translocation Independently of p53 and Retinoblastoma Status. Molecular Pharmacology.

[27] Chen Q, Xie W, Kuhn DJ, Voorhees PM, Lopez-Girona A, Mendy D, Corral LG (2008). Targeting the p27 E3 Ligase SCF(Skp2) Results in p27- and Skp2-Mediated Cell-Cycle Arrest and Activation of Autophagy. Blood.

[28] Komata T, Kanzawa T, Takeuchi H, Germano IM, Schreiber M, Kondo Y, Kondo S (2003). Antitumour Effect of Cyclin-Dependent Kinase Inhibitors (p16(INK4A), p18(INK4C), p19(INK4D), p21(WAF1/CIP1) and p27(KIP1)) on Malignant Glioma Cells. British Journal of Cancer.

[29] Liang J, Shao SH, Xu ZX, Hennessy B, Ding Z, Larrea M, Kondo S, Dumont DJ, Gutterman JU, Walker CL, Slingerland JM, Mills GB (2007). The Energy Sensing LKB1-AMPK Pathway Regulates p27(kip1) Phosphorylation Mediating the Decision to Enter Autophagy or Apoptosis. Nature Cell Biology.

[30] Liu YP, Chen HL, Tzeng CC, Lu PJ, Lo CW, Lee YC, Tseng CH (2013). TCH-1030 Targeting on Topoisomerase I Induces S-Phase Arrest, DNA Fragmentation, and Cell Death Of Breast Cancer Cells. Breast Cancer Research and Treatment.

[31] Yoo SH, Yoon YG, Lee JS, Song YS, Oh JS, Park BS, Yoo YH (2012). Etoposide Induces a Mixed Type of Programmed Cell Death and Overcomes the Resistance Conferred by Bcl-2 in Hep3B Hepatoma Cells. International Journal of Oncology.

[32] Schleich K, Krammer PH, Lavrik IN (2013). The Chains of Death: A New View on Caspase-8 Activation at the DISC. Cell Cycle.

[33] Ehrhardt H, Häcker S, Wittmann S, Maurer M, Borkhardt A, Toloczko A, Debatin KM, Fulda S, Jeremias I (2008). Cytotoxic Drug-Induced, p53-Mediated Upregulation of Caspase-8 in Tumor Cells. Oncogene.

[34] Shawgo ME, Shelton SN, Robertson JD (2008). Caspase-Mediated Bak Activation and Cytochrome C Release during Intrinsic Apoptotic Cell Death in Jurkat Cells. The Journal of Biological Chemistry.

[35] Enari M, Sakahira H, Yokoyama H, Okawa K, Iwamatsu A, Nagata S (1998). A Caspase-Activated DNase that Degrades DNA during Apoptosis, and Its Inhibitor ICAD. Nature.

[36] Alenzi FQ, Lotfy M, Wyse R (2010). Swords of Cell Death: Caspase Activation and Regulation. Asian Pacific Journal of Cancer Prevention: APJCP.

[37] Liu J, Uematsu H, Tsuchida N, Ikeda MA (2011). Essential Role of Caspase-8 in p53/p73-Dependent Apoptosis Induced by Etopo Side in Head and Neck Carcinoma Cells. Molecular Cancer.

[38] Hitotsuyanagi Y, Kobayashi M, Morita H, Itokawa H, Takeya K (1999). Synthesis of (-)-4-Aza-4-deoxypodophyllotoxin from (-)-Podophyllotoxin. Tetrahedron Letters.

[39] Hitotsuyanagi Y, Fukuyo M, Tsuda K, Kobayashi M, Ozeki A, Itokawa H, Takeya K (2000). 4-Aza-2,3-dehydro-4-deoxypodophyllotoxins: Simple Aza-Podophyllotoxin Analogues Possessing Potent Cytotoxicity. Bioorganic & Medicinal Chemistry Letters.

[40] Raimond BG, Ravelli BG, Patrick AC, Isabelle J, Sylvie L, André S, Marcel K (2004). Insight into Tubulin Regulation from a Complex with Colchicine and a Stathmin-Like Domain. Nature.

